# Non-steroidal anti-inflammatory drug use and outcomes of COVID-19 in the ISARIC Clinical Characterisation Protocol UK cohort: a matched, prospective cohort study

**DOI:** 10.1016/S2665-9913(21)00104-1

**Published:** 2021-05-07

**Authors:** Thomas M Drake, Cameron J Fairfield, Riinu Pius, Stephen R Knight, Lisa Norman, Michelle Girvan, Hayley E Hardwick, Annemarie B Docherty, Ryan S Thwaites, Peter J M Openshaw, J Kenneth Baillie, Ewen M Harrison, Malcolm G Semple, ISARIC4C Investigators, J Kenneth Baillie, Malcolm G Semple, Peter JM Openshaw, Gail Carson, Beatrice Alex, Benjamin Bach, Wendy S Barclay, Debby Bogaert, Meera Chand, Graham S Cooke, Ana da Silva Filipe, Thushan de Silva, Annemarie B Docherty, Jake Dunning, Tom Fletcher, Christopher A Green, Ewen M Harrison, Julian A Hiscox, Antonia YW Ho, Peter W Horby, Samreen Ijaz, Say Khoo, Paul Klenerman, Andrew Law, Wei Shen Lim, Alexander J Mentzer, Laura Merson, Alison M Meynert, Shona C Moore, Mahdad Noursadeghi, Massimo Palmarini, William A Paxton, Georgios Pollakis, Nicholas Price, Andrew Rambaut, David L Robertson, Clark D Russell, Vanessa Sancho-Shimizu, Janet T Scott, Louise Sigfrid, Tom Solomon, Shiranee Sriskandan, David Stuart, Charlotte Summers, Richard S Tedder, AA Roger Thompson, Emma C Thomson, Ryan S Thwaites, Lance CW Turtle, Maria Zambon, Chloe Donohue, Fiona Griffiths, Hayley Hardwick, Ruth Lyons, Wilna Oosthuyzen, Thomas M Drake, Cameron J Fairfield, Stephen R Knight, Kenneth A Mclean, Derek Murphy, Lisa Norman, Riinu Pius, Catherine A Shaw, Marie Connor, Jo Dalton, Carrol Gamble, Michelle Girvan, Sophie Halpin, Janet Harrison, Clare Jackson, Laura Marsh, Stephanie Roberts, Egle Saviciute, Sara Clohisey, Ross Hendry, Andrew Law, Gary Leeming, James Scott-Brown, Murray Wham, William Greenhalf, Sara McDonald, Victoria Shaw, Seán Keating, Katie A. Ahmed, Jane A Armstrong, Milton Ashworth, Innocent G Asiimwe, Siddharth Bakshi, Samantha L Barlow, Laura Booth, Benjamin Brennan, Katie Bullock, Nicola Carlucci, Emily Cass, Benjamin WA Catterall, Jordan J Clark, Emily A Clarke, Sarah Cole, Louise Cooper, Helen Cox, Christopher Davis, Oslem Dincarslan, Alejandra Doce Carracedo, Chris Dunn, Philip Dyer, Angela Elliott, Anthony Evans, Lorna Finch, Lewis WS Fisher, Lisa Flaherty, Terry Foster, Isabel Garcia-Dorival, William Greenhalf, Philip Gunning, Catherine Hartley, Anthony Holmes, Rebecca L Jensen, Christopher B Jones, Trevor R Jones, Shadia Khandaker, Katharine King, Robyn T. Kiy, Chrysa Koukorava, Annette Lake, Suzannah Lant, Diane Latawiec, Lara Lavelle-Langham, Daniella Lefteri, Lauren Lett, Lucia A Livoti, Maria Mancini, Hannah Massey, Nicole Maziere, Sarah McDonald, Laurence McEvoy, John McLauchlan, Soeren Metelmann, Nahida S Miah, Joanna Middleton, Joyce Mitchell, Shona C Moore, Ellen G Murphy, Rebekah Penrice-Randal, Jack Pilgrim, Tessa Prince, Will Reynolds, P. Matthew Ridley, Debby Sales, Victoria E Shaw, Rebecca K Shears, Benjamin Small, Krishanthi S Subramaniam, Agnieska Szemiel, Aislynn Taggart, Jolanta Tanianis-Hughes, Jordan Thomas, Erwan Trochu, Libby van Tonder, Eve Wilcock, J. Eunice Zhang, Alan MacLean, Sarah McCafferty, Kirstie Morrice, Lee Murphy, Nicola Wrobel, Kayode Adeniji, Daniel Agranoff, Ken Agwuh, Dhiraj Ail, Erin L. Aldera, Ana Alegria, Brian Angus, Abdul Ashish, Dougal Atkinson, Shahedal Bari, Gavin Barlow, Stella Barnass, Nicholas Barrett, Christopher Bassford, Sneha Basude, David Baxter, Michael Beadsworth, Jolanta Bernatoniene, John Berridge, Nicola Best, Pieter Bothma, Robin Brittain-Long, Naomi Bulteel, Tom Burden, Andrew Burtenshaw, Vikki Caruth, David Chadwick, David Chadwick, Duncan Chambler, Nigel Chee, Jenny Child, Srikanth Chukkambotla, Tom Clark, Paul Collini, Catherine Cosgrove, Jason Cupitt, Maria-Teresa Cutino-Moguel, Paul Dark, Chris Dawson, Samir Dervisevic, Phil Donnison, Sam Douthwaite, Ingrid DuRand, Ahilanadan Dushianthan, Tristan Dyer, Cariad Evans, Chi Eziefula, Chrisopher Fegan, Adam Finn, Duncan Fullerton, Sanjeev Garg, Sanjeev Garg, Atul Garg, Effrossyni Gkrania-Klotsas, Jo Godden, Arthur Goldsmith, Clive Graham, Elaine Hardy, Stuart Hartshorn, Daniel Harvey, Peter Havalda, Daniel B Hawcutt, Maria Hobrok, Luke Hodgson, Anil Hormis, Michael Jacobs, Susan Jain, Paul Jennings, Agilan Kaliappan, Vidya Kasipandian, Stephen Kegg, Michael Kelsey, Jason Kendall, Caroline Kerrison, Ian Kerslake, Oliver Koch, Gouri Koduri, George Koshy, Shondipon Laha, Steven Laird, Susan Larkin, Tamas Leiner, Patrick Lillie, James Limb, Vanessa Linnett, Jeff Little, Mark Lyttle, Michael MacMahon, Emily MacNaughton, Ravish Mankregod, Huw Masson, Elijah Matovu, Katherine McCullough, Ruth McEwen, Manjula Meda, Gary Mills, Jane Minton, Mariyam Mirfenderesky, Kavya Mohandas, Quen Mok, James Moon, Elinoor Moore, Patrick Morgan, Craig Morris, Katherine Mortimore, Samuel Moses, Mbiye Mpenge, Rohinton Mulla, Michael Murphy, Thapas Nagarajan, Megan Nagel, Mark Nelson, Matthew K. O'Shea, Marlies Ostermann, Igor Otahal, Mark Pais, Selva Panchatsharam, Danai Papakonstantinou, Padmasayee Papineni, Hassan Paraiso, Brij Patel, Natalie Pattison, Justin Pepperell, Mark Peters, Mandeep Phull, Stefania Pintus, Frank Post, David Price, Rachel Prout, Nikolas Rae, Henrik Reschreiter, Tim Reynolds, Neil Richardson, Mark Roberts, Devender Roberts, Alistair Rose, Guy Rousseau, Brendan Ryan, Taranprit Saluja, Sarah Sarah, Aarti Shah, Manu Shankar-Hari, Prad Shanmuga, Anil Sharma, Anna Shawcross, Jagtur Singh Pooni, Jeremy Sizer, Richard Smith, Catherine Snelson, Nick Spittle, Nikki Staines, Tom Stambach, Richard Stewart, Pradeep Subudhi, Tamas Szakmany, Kate Tatham, Jo Thomas, Chris Thompson, Robert Thompson, Ascanio Tridente, Darell Tupper-Carey, Mary Twagira, Andrew Ustianowski, Nick Vallotton, Lisa Vincent-Smith, Shico Visuvanathan, Alan Vuylsteke, Sam Waddy, Rachel Wake, Andrew Walden, Ingeborg Welters, Tony Whitehouse, Paul Whittaker, Ashley Whittington, Meme Wijesinghe, Martin Williams, Lawrence Wilson, Stephen Winchester, Martin Wiselka, Adam Wolverson, Daniel G Wooton, Andrew Workman, Bryan Yates, Peter Young

**Affiliations:** a Centre for Medical Informatics, Usher Institute, University of Edinburgh, Edinburgh, UK; b Liverpool Clinical Trials Centre, University of Liverpool, Liverpool, UK; c Health Protection Research Unit in Emerging and Zoonotic Infections, Institute of Infection, Veterinary and Ecological Sciences, Faculty of Health and Life Sciences, University of Liverpool, Liverpool, UK; d National Heart and Lung Institute, Imperial College London, UK; e Roslin Institute, University of Edinburgh, Easter Bush Campus, Edinburgh, UK; f Department of Respiratory Medicine, Alder Hey Children's Hospital, Liverpool, UK

## Abstract

**Background:**

Early in the pandemic it was suggested that pre-existing use of non-steroidal anti-inflammatory drugs (NSAIDs) could lead to increased disease severity in patients with COVID-19. NSAIDs are an important analgesic, particularly in those with rheumatological disease, and are widely available to the general public without prescription. Evidence from community studies, administrative data, and small studies of hospitalised patients suggest NSAIDs are not associated with poorer COVID-19 outcomes. We aimed to characterise the safety of NSAIDs and identify whether pre-existing NSAID use was associated with increased severity of COVID-19 disease.

**Methods:**

This prospective, multicentre cohort study included patients of any age admitted to hospital with a confirmed or highly suspected SARS-CoV-2 infection leading to COVID-19 between Jan 17 and Aug 10, 2020. The primary outcome was in-hospital mortality, and secondary outcomes were disease severity at presentation, admission to critical care, receipt of invasive ventilation, receipt of non-invasive ventilation, use of supplementary oxygen, and acute kidney injury. NSAID use was required to be within the 2 weeks before hospital admission. We used logistic regression to estimate the effects of NSAIDs and adjust for confounding variables. We used propensity score matching to further estimate effects of NSAIDS while accounting for covariate differences in populations.

**Results:**

Between Jan 17 and Aug 10, 2020, we enrolled 78 674 patients across 255 health-care facilities in England, Scotland, and Wales. 72 179 patients had death outcomes available for matching; 40 406 (56·2%) of 71 915 were men, 31 509 (43·8%) were women. In this cohort, 4211 (5·8%) patients were recorded as taking systemic NSAIDs before admission to hospital. Following propensity score matching, balanced groups of NSAIDs users and NSAIDs non-users were obtained (4205 patients in each group). At hospital admission, we observed no significant differences in severity between exposure groups. After adjusting for explanatory variables, NSAID use was not associated with worse in-hospital mortality (matched OR 0·95, 95% CI 0·84–1·07; p=0·35), critical care admission (1·01, 0·87–1·17; p=0·89), requirement for invasive ventilation (0·96, 0·80–1·17; p=0·69), requirement for non-invasive ventilation (1·12, 0·96–1·32; p=0·14), requirement for oxygen (1·00, 0·89–1·12; p=0·97), or occurrence of acute kidney injury (1·08, 0·92–1·26; p=0·33).

**Interpretation:**

NSAID use is not associated with higher mortality or increased severity of COVID-19. Policy makers should consider reviewing issued advice around NSAID prescribing and COVID-19 severity.

**Funding:**

National Institute for Health Research and Medical Research Council.

## Introduction

Non-steroidal anti-inflammatory drugs (NSAIDs) provide effective analgesia and are important in the treatment of inflammatory diseases. They form a part of the WHO pain ladder and have opioid-sparing properties, supported by data from randomised trials.^1^ In March, 2020, the French health ministry and media discussed unpublished data showing that use of NSAIDs could increase the severity of COVID-19.^2^, ^3^ Debate ensued, with some arguing that NSAIDs should be avoided as a result of these findings.^3^, ^4^, ^5^ This debate led to several regulatory authorities calling for urgent investigation of NSAIDs and COVID-19 severity.^6^

More recent studies have found no associations between NSAID use, admission to hospital, and worse outcomes for patients with COVID-19.^7^, ^8^, ^9^, ^10^, ^11^, ^12^, ^13^ These studies have been done in several different populations. In the community, administrative data have not shown an increased risk of hospitalisation for patients with COVID-19 taking NSAIDs.^7^, ^11^, ^13^ Data on patients admitted to hospital with COVID-19 are more scarce but suggest that patients taking NSAIDs do not have poorer outcomes compared with not taking NSAIDs.^10^, ^11^, ^12^ Studies that focus on cohorts of hospitalised patients with COVID-19 have included participants from single centres or included only small numbers of patients taking NSAIDs.

Studies of patients with non-SARS-CoV-2 respiratory infection have found associations between NSAID (including cyclooxygenase [COX]-2 inhibitors) use and increased rates of complications.^14^, ^15^, ^16^, ^17^, ^18^, ^19^ These studies found that NSAID use was associated with higher rates of myocardial infarction, pleural empyema, and longer length of hospital stay. However, outcomes used in such pneumonia studies, for example empyema, are less frequent in patients with SARS-CoV-2 infection. There are recognised safety concerns with the use of NSAIDs, including increased incidence of stroke, gastrointestinal bleeding, myocardial infarction, acute kidney injury, and bleeding,^14^, ^15^, ^16^, ^17^, ^20^ which are more common in older people.

**Research in context**

**Evidence before this study**

There have been anecdotal reports that use of non-steroidal anti-inflammatory drugs (NSAIDs) is linked to COVID-19 severity and poor outcomes. NSAIDs are an important analgesic class, used in the management of acute pain and rheumatological diseases. We searched PubMed from inception to Jan 12, 2021, using the terms “NSAIDs” and “COVID-19”, with no language restrictions. Several studies, in various populations, have identified that patients taking NSAIDs who contract SARS-CoV-2 infection are not at higher risk of admission to hospital or death. However, the populations included in these studies are frequently small, based on routine administrative data, or are drawn from community populations and hence have relatively low rates of SARS-CoV-2 infection.

**Added value of this study**

This prospective, multicentre study at 255 UK healthcare facilities found that in patients who were admitted to hospital with COVID-19, those taking NSAIDs before admission had the same outcomes as those who did not. We did not find any differences in mortality or disease severity, or in secondary outcomes including admission to critical care, use of ventilation, use of oxygen, or presence of acute kidney injury.

**Implications of all the available evidence**

Those taking NSAIDs do not appear to have poorer COVID-19 outcomes. To our knowledge, our prospective study includes the largest number of patients admitted to hospital with COVID-19 to date, and adds to the literature on the safety of NSAIDs and in-hospital outcomes. NSAIDs do not appear to increase the risk of worse in-hospital outcomes. NSAIDs are an important analgesic modality and have a vital opioid-sparing role in pain management. Patients and clinicians should be reassured by these findings that NSAIDs are safe in the context of the pandemic.

By contrast, a randomised trial in the UK found that ibuprofen reduced the symptom severity of acute respiratory tract infection in patients in the community.^21^ In preclinical models, there is evidence that NSAIDs decrease pulmonary oedema, lessen endothelial leakiness, and reduce the severity of acute respiratory distress syndrome (ARDS), leading to the suggestion they might be useful in the treatment of COVID-19, with at least one clinical trial currently underway.^22^, ^23^, ^24^

We aimed to characterise the safety of NSAIDs and identify whether pre-existing NSAID use was associated with increased severity of COVID-19 disease.

## Methods

### Study design and participants

The International Severe Acute Respiratory and Emerging Infection Consortium (ISARIC) Clinical Characterisation Protocol (CCP) for Severe Emerging Infection was developed in 2009 and activated in response to the SARS-CoV-2 pandemic on Jan 17, 2020. ISARIC-CCP-UK is an actively recruiting prospective cohort study across England, Scotland, and Wales. The protocol, revision history, case report forms, study information, and consent forms are available online. ISARIC CCP UK received ethical approval from the South Central—Oxford C Research Ethics Committee in England (13/SC/0149) and by the Scotland A Research Ethics Committee (20/SS/0028). As required, patients gave written informed consent. The study is reported in line with the STROBE guidelines.^25^

Patients of any age admitted to hospital with a confirmed or highly suspected SARS-CoV-2 infection leading to COVID-19 between Jan 17 and Aug 10, 2020, were eligible for inclusion in the study. Confirmation of SARS-CoV-2 infection was by reverse transcription-PCR, which was the only testing method available in the UK during the reported study period. Highly suspected cases were eligible for inclusion, given that SARS-CoV-2 was an emergent pathogen at time of protocol activation. We excluded patients who did not have death or discharge outcomes available.

### Procedures

Data were collected by clinical research staff using a standardised case report form and entered into a Research Electronic Data Capture secure online database.^26^ Data were captured across multiple timepoints, including admission, hospital stay (days 1, 3, 6, and 9), and discharge. Characteristics captured included age, sex, asthma, chronic cardiac disease, chronic haematological disease, chronic kidney disease, chronic neurological disease, chronic non-asthmatic pulmonary disease, HIV/AIDs, malignancy, liver disease, obesity, rheumatological disorder, and smoking history. Physiological parameters at admission were captured, including components of the National Early Warning Score 2 (NEWS2) and the quick Sequential Organ Failure Assessment (qSOFA).

Current medication or medication taken within the past 2 weeks was recorded on hospital admission. The NSAID group was defined as patients taking generic or branded NSAIDs available within the UK, determined using the NHS Technology Reference data update distribution service, which were mapped to entered drug names within the study database. We defined exposure to NSAIDs as patients taking non-selective COX inhibitors or COX-2 specific inhibitors. Topical NSAID preparations were excluded. Aspirin was not considered an NSAID for the purposes of this analysis, as aspirin is frequently used for the treatment and prevention of conditions which are different to those for which NSAIDs are indicated.

### Outcomes

The primary outcome was in-hospital mortality (including palliative discharge). Secondary outcomes were admission to critical care (level 3 intensive care unit or level 2 high dependency unit), use of invasive mechanical ventilation, use of non-invasive ventilation, use of supplementary oxygen, and occurrence of acute kidney injury. Acute kidney injury was defined according to the Kidney Disease: Improving Global Outcomes guidelines.^27^ We followed up patients for the duration of their hospital admission. Patients who were admitted after Aug 3, 2020, were excluded to avoid bias from patients with a long hospital stay or who had not had adequate time to accrue secondary outcomes.

### Statistical analysis

Categorical data are presented as frequencies and percentages. Normally distributed variables are summarised as mean (SD) and non-normally distributed variables as median (IQR). χ^2^ test was used to compare categorical data, except where expected cell counts were five or fewer, in which case Fisher's exact test was used. Continuous variables were compared using Welch's *t*-test or the Kruskall-Wallis test, depending on the distribution of data.

We used propensity score matching to estimate the treatment effect of NSAIDs while accounting for covariate imbalance, using a multistep approach. First, multiple imputation by chained equations was done using available explanatory variables (age, sex, diabetes [type 1 or type 2], chronic cardiac disease, chronic renal disease, obesity, chronic pulmonary disease, ethnicity, dementia, and rheumatological disease) and outcomes (five imputed datasets with five iterations per dataset; distributions checked graphically, and convergence confirmed). Second, logistic regression was used to determine the log odds of NSAID use (propensity scores) using the variables stated above. For logistic regression models, patient-level explanatory variables were entered as fixed effects and in unmatched models, hospital was used as a random effect. We did not use random effects for matched models to ensure we could match on clinical characteristics, rather than restrict matches to within each centre. Following this, propensity score matching was done within each imputed dataset, and patients taking NSAIDs were matched (1:1) with their nearest neighbour not taking NSAIDs.^28^ Balance was determined using standardised mean differences. Fourth, effects estimates were determined, and results were pooled using Rubin's rules.^29^ Effect estimates are presented as odds ratios (ORs) for binary outcome data, with corresponding 95% CIs. Imputed and matched data are presented as pooled models.

For unmatched models, clinically plausible variables associated with NSAID use and clinical outcomes were incorporated into the modelling approach. These variables included age, sex, and presence of chronic cardiac disease, chronic pulmonary disease, diabetes, obesity, chronic renal disease, rheumatological disease, and dementia. First order interactions were checked before final model selection, which was guided by minimisation of the Akaike Information Criterion. p<0·05 was considered to indicate a statistically significant difference.

We did four separate sensitivity analyses. First, we included patients taking non-ibuprofen NSAIDs only, as these usually require a prescription in the UK and are more likely to be taken for longer periods than ibuprofen. Next, we did an analysis including patients who were admitted at least 7 days after symptom onset to investigate whether NSAID use had any effect in those without nosocomial infection. We then did an analysis confined to patients with rheumatic disease, as this group are likely to be on long-term NSAID treatment compared with individuals who might be taking NSAIDs for short-term analgesia. Finally, to ensure the secondary outcomes were robust and to establish whether death was likely to compete with these outcomes, we did three sensitivity analyses to see if death altered the direction or magnitude of the effect size. For the first sensitivity analysis we excluded those who died. For the second sensitivity analysis we used deterioration (death or requirement for critical care) as the outcome. Lastly, for the third sensitivity analysis, we looked at mortality by NSAID use only in those not admitted to critical care.

Data were analysed using R version 3.6.3, using the tidyverse, finalfit, mice, MatchThem, cobalt, and matchit packages.

### Role of the funding source

The funders of the study had no role in study design, data collection, data analysis, data interpretation, or writing of the report.

## Results

**Figure 1:**
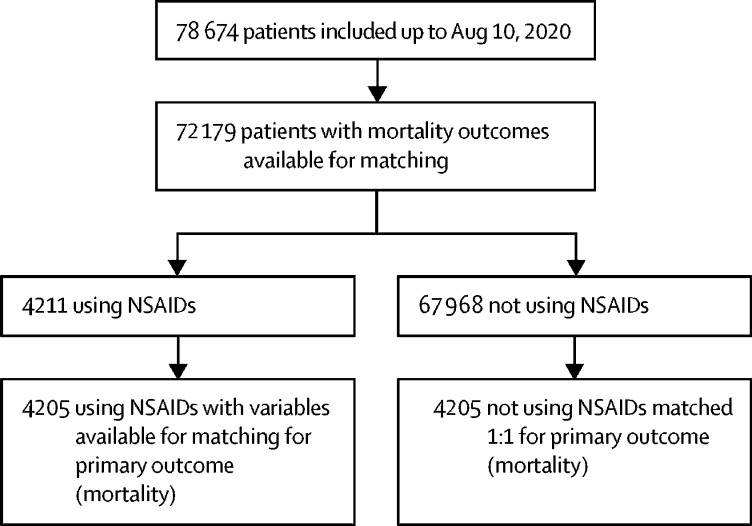
Study profile

Between Jan 17 and Aug 10, 2020, we enrolled 78 674 patients across 255 health-care facilities in England, Scotland, and Wales (figure 1), representing around 60% of the total number of people admitted to hospital with COVID-19 over that time period. 72 179 patients had death outcomes available for matching. We observed no large differences in distribution of explanatory variables by missing mortality outcome (appendix p 1). In this cohort, 4211 (5·8%) patients were recorded as taking systemic NSAIDs before admission to hospital. In the unmatched data, patients who received NSAIDs were more likely to be female, and significantly more likely to have pre-existing rheumatological disease (table 1; appendix p 2). Propensity score matching produced balanced, well-matched treatment groups for each set of imputed and pooled models (appendix pp 3–8, 13).

**Table 1:** Unmatched patient characteristics by NSAID use

		**No NSAIDs (N=67 968)**	**NSAIDs (N=4211)**	**p value**
Age at admission, years (n=71 987)		70·2 (18·4)	70·1 (18.7)	0·765*
Sex (n=71 915)		..	..	0·0008
	Male	38 151 (56·1%)	2255 (53·6%)	..
	Female	29 564 (43·5%)	1945 (46·2%)	..
	Missing	253 (0·4%)	11 (0·3%)	..
Ethnicity (n=64 123)		..	..	0·116
	Asian	3708 (5·5%)	230 (5·5%)	..
	Black	2358 (3·5%)	118 (2·8%)	..
	White	50 124 (73·7%)	3109 (73·8%)	..
	Other	4201 (6·2%)	275 (6·5%)	..
	Missing	7577 (11·1%)	479 (11·4%)	..
Smoking status (n=43 585)		..	..	0·0001
	Current smoker	3588 (5·3%)	228 (5·4%)	..
	Never smoked	22 896 (33·7%)	1394 (33·1%)	..
	Former smoker	14 428 (21·2%)	1051 (25·0%)	..
	Missing	27 056 (39·8%)	1538 (36·5%)	..
Chronic cardiac disease (n=67 454)		..	..	<0·0001
	No	42 831 (63·0%)	2557 (60·7%)	..
	Yes	20 588 (30·3%)	1478 (35·1%)	..
	Missing	4549 (6·7%)	176 (4·2%)	..
Chronic kidney disease (n=66 964)		..	..	0·042
	No	51 800 (76·2%)	3237 (76·9%)	..
	Yes	11 167 (16·4%)	760 (18·0%)	..
	Missing	5001 (7·4%)	214 (5·1%)	..
Chronic pulmonary disease (not asthma; n=67 171)		..	..	0·0030
	No	51 933 (76·4%)	3219 (76·4%)	..
	Yes	11 232 (16·5%)	787 (18·7%)	..
	Missing	4803 (7·1%)	205 (4·9%)	..
Obesity (as defined by clinical staff; n=60 199)		..	..	<0·0001
	No	49 993 (73·6%)	3039 (72·2%)	..
	Yes	6590 (9·7%)	577 (13·7%)	..
	Missing	11 385 (16·8%)	595 (14·1%)	..
Diabetes (n=65 135)		..	..	0·189
	No diabetes	46 728 (68·8%)	2881 (68·4%)	
	Diabetes with complications	4484 (6·6%)	299 (7·1%)	..
	Diabetes without complications	10 150 (14·9%)	593 (14·1%)	..
	Missing	6606 (9·7%)	438 (10·4%)	..
Rheumatological disorder (n=66 228)		..	..	<0·0001
	No	55 469 (81·6%)	3145 (74·7%)	..
	Yes	6809 (10·0%)	805 (19·1%)	..
	Missing	5690 (8·4%)	261 (6·2%)	..
Dementia (n=66 788)		..	..	0·0003
	No	51 980 (76·5%)	3368 (80·0%)	..
	Yes	10 845 (16·0%)	595 (14·1%)	..
	Missing	5143 (7·6%)	248 (5·9%)	..

Data are mean (SD) or n (%). NSAID=Non-steroidal anti-inflammatory drug.

* Welch's two-sample *t*-test used.

1279 (30·4%) of 4211 patients in the NSAID group died versus 21 256 (31·3%) of 67 698 patients in in the no NSAIDs group (table 2; appendix p 14). In the unmatched cohort, in-hospital mortality was no different between NSAID users and non-users (table 2). After matching, NSAID use was not associated with worse in-hospital mortality (matched OR 0·95, 95% CI 0·84–1·07; p=0·35; table 3).

**Table 2:** Unmatched outcomes by NSAID use

		**No NSAIDs (N=67 968)**	**NSAIDs (N=4211)**	**p value**
Mortality (n=72 179)		..	..	0·227
	No	46 712 (68·7%)	2932 (69·6%)	..
	Yes	21 256 (31·3%)	1279 (30·4%)	..
Critical care admission (n=70 955)		..	..	0·467
	No	57507 (86.1%)	3599 (85.7%)	..
	Yes	9250 (13.9%)	599 (14.3%)	..
Invasive ventilation (n=69 972)		..	..	0·396
	No	60 254 (91·5%)	3821 (91·9%)	..
	Yes	5562 (8·5%)	335 (8·1%)	..
Non-invasive ventilation (n=69 818)		..	..	0·0047
	No	55 809 (85·0%)	3452 (83·3%)	..
	Yes	9867 (15·0%)	690 (16·7%)	..
Supplemental oxygen (n=70 124)		..	..	0·62
	No	22 826 (34·6%)	1420 (34·2%)	..
	Yes	43 147 (65·4%)	2731 (65·8%)	..
Acute kidney injury (n=68 228)		..	..	0·034
	No	48 258 (75·1%)	2945 (73·6)	..
	Yes	15 970 (24·9%)	1055 (26·4)	..

NSAID=Non-steroidal anti-inflammatory drug.

**Table 3:** Outcomes after propensity score matching between those using NSAIDs before admission and those not using NSAIDs

		**Effect estimate**	**p value**
**In-hospital mortality**			
No NSAIDs		1 (ref)	..
NSAIDs (n=4205)		0·95 (0·84 to 1·07)	0·35
**Secondary outcomes**			
No NSAIDs		1 (ref)	..
NSAIDs			
	Critical care admission (n=4198)	1·01 (0·87 to 1·17)	0·89
	Invasive ventilation (n=4156)	0·96 (0·80 to 1·17)	0·69
	Non-invasive ventilation (n=4142)	1·12 (0·96 to 1·32)	0·14
	Oxygen (n=4151)	1·00 (0·89 to 1·12)	0·97
	Acute kidney injury (n=4000)	1·08 (0·92 to 1·26)	0·33
**Severity on admission**			
Physiological scores			
	qSOFA score (n=3793)	−0·02 (−0·06 to 0·02)	0·42
	NEWS2 (n=3721)	−0·08 (−0·30 to 0·14)	0·46
Physiological parameters			
	Heart rate (n=4102)	−0·40 (−1·39 to 0·59)	0·43
	Respiratory rate (n=4096)	−0·17 (−0·66 to 0·32)	0·48
	Saturation of peripheral oxygen (n=4076)	−0·00 (−0·27 to 0·26)	0·98
	Systolic blood pressure (n=4085)	1·09 (−0·07 to 2·25)	0·066
	Diastolic blood pressure (n=4071)	−0·21 (−0·93 to 0·51)	0·56

Effect estimates are either matched odds ratio (95% CI) or mean difference (95% CI). NSAID=non-steroidal anti-inflammatory drug. NEWS2=National Early Warning Score 2. qSOFA=quick Sequential Organ Failure Assessment.

In a sensitivity analysis of patients admitted to hospital at least 7 days after symptom onset (19 734 [27·3%] of 72 179 patients) who were taking NSAIDs matched to patients not taking NSAIDs who presented during the same timeframe, we found no difference in mortality (matched OR 1·11, 95% CI 0·88–1·39; p=0·37). In patients with rheumatological disease (7614 [10·5%] of 72 179), use of NSAIDs was not associated with increased mortality (matched OR 0·90, 0·68–1·19; p=0·44).

**Figure 2:**
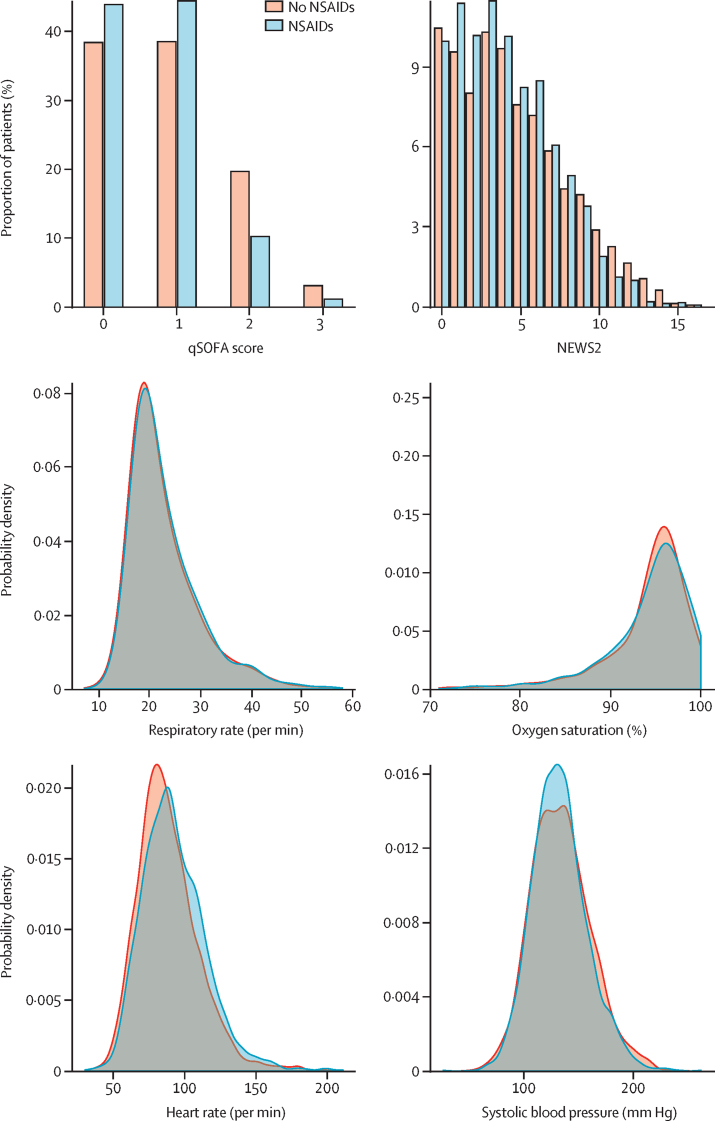
Physiological parameters on admission to hospital in NSAID users and those not taking NSAIDs NSAID=non-steroidal anti-inflammatory drug. NEWS2=National Early Warning Score 2. qSOFA=quick Sequential Organ Failure Assessment.

In the unmatched cohort, NSAID users were more likely to require non-invasive ventilation and sustain acute kidney injury (table 2). After matching, those taking NSAIDs were no more likely to require critical care admission (1·01, 0·87–1·17; p=0·89), invasive ventilation (0·96, 0·80–1·17; p=0·69), non-invasive ventilation (1·12, 0·96–1·32; p=0·14), or supplementary oxygen (1·00, 0·89–1·12; p=0·97), or to sustain acute kidney injury (1·08, 0·92–1·26; p=0·33), compared with those not taking NSAIDs (table 3; appendix p 14). In addition, on admission to hospital, matched patients on NSAIDs had similar qSOFA and NEWS2 scores to those who did not receive NSAIDs (table 3; figure 2). When we did a sensitivity analysis, excluding those who died, our findings did not change, and we did not observe an increase or decrease in associations between NSAIDs and any of the secondary outcomes (appendix p 9). We did a further two sensitivity analyses to ensure the secondary outcomes were robust. First, we combined death and critical care outcomes. Second, we looked at mortality in the population who did not require critical care. These analyses showed no association between NSAIDs and the chances of death or admission to critical care when these outcomes were combined (OR 0·94, 95% CI 0·83–1·06; p=0·28), nor any association with death in patients who were not admitted to critical care (0·92, 0·82–1·03; p=0·16).

The most common NSAID used was ibuprofen, followed by other NSAIDs—eg, diclofenac, ketorolac, naproxen, oxicams—and COX-2 inhibitors. We found no significant differences in mortality by type of NSAID (appendix p 10). We created matched groups to compare ibuprofen with no NSAID use, and ibuprofen with other NSAIDs, as a sensitivity analysis to explore whether NSAIDs associated with longer-term use had a different safety profile compared with ibuprofen. Use of ibuprofen was not significantly associated with increased mortality compared with those not taking NSAIDs (matched OR 0·90, 95% CI 0·71–1·13; p=0·36; appendix p 11) or any other NSAID (matched OR 0·82, 0·66–1·03; p=0·082; appendix p 12).

## Discussion

In this study, patients admitted to hospital with COVID-19 who were taking NSAIDs did not have more severe disease than did patients who were not taking NSAIDs. Mortality, critical care admission, respiratory support, and acute kidney injury were also not significantly different across matched NSAID treatment groups. We found no evidence of harm caused by NSAID use in patients admitted to hospital with severe COVID-19.

Early on in the COVID-19 pandemic, questions were raised concerning the safety of NSAIDs in patients with COVID-19, with suggestions that these drugs were leading to more severe disease in a some patients.^2^, ^30^, ^31^ Our data show that patients taking NSAIDs did not have more severe symptoms or poorer outcomes than those not taking NSAIDs. These data support community studies showing that NSAID users did not have higher rates of hospitalisation with COVID-19 and smaller studies of in-hospital outcomes, which found NSAID use was not associated with poorer outcomes. A propensity matched data linkage study of patients with osteoarthritis taking NSAIDs in the community setting found no difference in the risk of developing COVID-19 or dying from the disease.^13^ Compared with our data and previous studies our consortium has published, this data linkage study^13^ did not find any differences in risk factors for mortality after COVID-19, which is probably due to the very small numbers of patients with COVID-19 in the study. To our knowledge, our study is the largest study of in-hospital outcomes of patients with COVID-19 to date. Considering all the evidence, if there was an extreme effect of NSAIDs on COVID-19 outcomes or severity, this would have been observed in one or more of the studies that have been done, including the present study.

To our knowledge, worldwide, this is the largest prospective study of patients admitted to hospital with COVID-19. We were able to collect real-time data on patients to study their outcomes and collect detailed comorbidity data. Clinical research staff collected data on medications that patients had been prescribed or were currently taking, or had been taking within the past 14 days. These data would otherwise be challenging to obtain from routine sources of health-care data. Although we have only captured data on patients admitted to hospital with COVID-19 that are available within the ISARIC CCP, this represents around 60% of all patients hospitalised with COVID-19 in the UK during the period of the study. We did not capture data for patients who had the disease in the community and did not require hospital admission, or who died in the community without hospital admission. Despite this, we expect that most patients who had severe COVID-19 would be admitted to hospital and thus captured in our dataset. A further potential limitation of our study is the absence of information on the indication for NSAIDs and duration of use. These missing data make it difficult to know whether individuals were taking NSAIDs for long-term conditions, or symptomatic relief for COVID-19 symptoms. Similarly, it is unclear whether patients continued taking NSAIDs during their inpatient admission. Therefore, we are unable to make any recommendations on whether NSAIDs should be continued after admission to hospital. To address this, we did a sensitivity analysis comparing use of ibuprofen to no NSAIDs or use of other non-ibuprofen NSAIDs, as ibuprofen use is most likely to be short-term. We observed no increase in poorer outcomes in those who used ibuprofen compared with those who did not use NSAIDs. Similarly, older patients, who are at greatest risk of adverse outcomes from COVID-19, might be less likely to be taking NSAIDs compared with other, more healthy and fit populations, as older patients with greater numbers of comorbidities are less likely to be prescribed NSAIDs because of their side-effect profile; therefore, our matching might not have incorporated this patient group fully. However, as older patients are less likely to be taking NSAIDs and the safety debate concerns younger populations, this is unlikely affect our results and their relevance to clinical practice.

There are several other important limitations to our study that must be considered. First, the most used NSAID was ibuprofen, which might not be generalisable to every country. Different NSAIDs are known to have different side-effect profiles; therefore, clinical trials of a specific compound might not be generalisable to an entire drug class.^32^ Additionally, our data did not contain information on drug dosages or adherence, so we were unable to model dose–response data. Second, although our study captured data on most patients hospitalised with COVID-19 in the UK during the period it was done, a few centres did not participate. However, our data is concordant with other datasets that focused on smaller populations within our study, such as data from the Intensive Care National Audit and Research Centre.^33^ Therefore, we consider our data to be meaningful and useful to help answer important clinical questions in patients with COVID-19. Another limitation is that to obtain the best possible matches for patients receiving NSAIDs, we did not include the date of admission as a matching variable. Mortality for patients admitted to hospital over the course of the pandemic has decreased, but this is unlikely to have affected our conclusions given that the time period we conducted our study during was limited largely to the first UK wave of infection. Finally, our data lack a non-SARS-CoV-2 comparator group to provide a temporal comparison with other critical illness or respiratory conditions. Future research could include a comparator group to investigate if NSAIDs modify or moderate outcomes of interest in patients with COVID-19 compared with other illnesses.

Although use of NSAIDs could, in theory, be beneficial in patients with COVID-19, we did not identify any evidence to support this. Clinical studies have suggested that release of proinflammatory mediators in COVID-19—including interleukin (IL)-1β, IL-6, and CCL2—is associated with more severe disease.^34^, ^35^ Preclinical studies in non-COVID-19 models have found that release of these cytokines can be inhibited by treatment with NSAIDs, leading to discussion around whether NSAIDs might be useful as a therapy for COVID-19.^23^, ^36^, ^37^ In these studies, NSAIDs have been shown to suppress IL-6 production and expression through various mechanisms, including suppression of prostaglandin E2, which upregulates production of IL-6 and IL-8.^36^, ^37^ Studies in bronchial epithelium have found that treatment with NSAIDs reduces expression of inflammatory mediators, including IL-6.^36^ A clinical trial of dexamethasone, which also has been shown to modulate inflammation,^38^ albeit probably through a separate mechanism, has been shown to reduce mortality in patients with COVID-19. Other immunomodulatory therapies are being trialled, including the IL-6 inhibitor tocilizumab. Results from the REMAP-CAP^39^ and RECOVERY^40^ trials showed that tocilizumab reduced the requirement for organ support and improved survival in patients with COVID-19, with further trials underway.^41^, ^42^ In addition to these trials, a randomised trial of ibuprofen in patients with COVID-19 is also underway.^23^

For clinicians and patients, our findings should provide reassurance that NSAIDs can be used as indicated in the community without increasing the severity of COVID-19. Our study did not capture whether NSAIDs were continued in hospital, so we cannot make any recommendations on whether these should be withheld or continued after hospital admission. There are important groups of patients who rely on NSAIDs for pain relief, including those with inflammatory joint diseases, bone pain, gout, postoperative pain, and menstrual pain, who would otherwise have few non-opioid options for pain relief. Taken together, clinicians should continue to prescribe and manage NSAIDs in the same way as before the COVID-19 pandemic began.

Future research in this area should focus on whether NSAIDs sufficiently modulate inflammation in COVID-19, by using both basic science and clinical approaches using appropriate outcomes that are directly measured. If benefit or harm is identified, finding the cellular mechanisms responsible for these effects will be important to inform the biological understanding of COVID-19. Finally, including groups that compare NSAIDs with alternative analgesics should be considered to provide evidence for clinicians and patients on the risks associated with alternative medications. In conclusion, policy makers should consider reviewing issued advice around NSAID prescribing and COVID-19 severity. NSAID use is not associated with poorer outcomes in patients admitted to hospital with COVID-19.

## Data sharing

Data, protocols, and all documentation around this analysis will be made available to academic researchers after authorisation from the independent data access and sharing committee. Data and analysis scripts are available on request to the Independent Data Management and Access Committee.

## Declaration of interests

All authors declare support from the National Institute for Health Research (NIHR), the Medical Research Council (MRC), the NIHR Health Protection Research Unit (HPRU) in Emerging and Zoonotic Infections at University of Liverpool, NIHR HPRU in Respiratory Infections at Imperial College London, NIHR Biomedical Research Centre (BRC) at Imperial College London, and NIHR Clinical Research Network for the submitted work. ABD reports grants from the UK Department of Health and Social Care (DHSC), during the conduct of the study, and grants from Wellcome Trust, outside the submitted work. PJMO reports personal fees from consultancies and from the European Respiratory Society, grants from MRC, MRC Global Challenge Research Fund, EU, NIHR BRC, MRC, GSK, Wellcome Trust, NIHR (Health Protection Research Unit [HPRU] in Respiratory Infection), and is NIHR senior investigator outside the submitted work. PJMO's role as President of the British Society for Immunology was unpaid but travel and accommodation at some meetings was provided. JKB reports grants from MRC. MGS reports grants from DHSC NIHR, MRC, and HPRU in Emerging and Zoonotic Infections, University of Liverpool, during the conduct of the study, and honoraria from Integrum Scientific, outside the submitted work. All other authors declare no support from any organisation for the submitted work, no financial relationships with any organisations that might have an interest in the submitted work in the previous 3 years, and no other relationships or activities that could appear to have influenced the submitted work.
