# Supplementary material for: Non-steroidal anti-inflammatory drug use and outcomes of COVID-19 in the ISARIC Clinical Characterisation Protocol UK cohort: a matched, prospective cohort study
Source: Lancet Rheumatol. 2021 May 7;3(7):e498–506. doi: 10.1016/S2665-9913(21)00104-1 (PMC8104907; doi:10.1016/S2665-9913(21)00104-1)
Supplement: Supplementary appendix [file mmc1.pdf]

THE LANCET
Rheumatology

Supplementary appendix

This appendix formed part of the original submission and has been peer reviewed. We post it as supplied by the authors.

Supplement to: Drake T M, Fairfield C J, Pius R, et al. Non-steroidal anti-inflammatory drug use and outcomes of COVID-19 in the ISARIC Clinical Characterisation Protocol UK cohort: a matched, prospective cohort study. *Lancet Rheumatol* 2021; **3:** e498–506.

Appendix

Table S1 – Missing data analysis

Missing data analysis: Death		Not missing	Missing
Age (years)	<50	9938 (13.8)	416 (16.7)
	50-69	19534 (27.1)	679 (27.3)
	70-79	16099 (22.4)	557 (22.4)
	80+	26419 (36.7)	838 (33.7)
Sex	Male	40406 (56.2)	1414 (56.4)
	Female	31509 (43.8)	1093 (43.6)
Chronic Cardiac Disease	No	45388 (67.3)	1147 (66.8)
	Yes	22066 (32.7)	569 (33.2)
Chronic Pulmonary Disease	No	55152 (82.1)	1382 (81.5)
	Yes	12019 (17.9)	314 (18.5)
Diabetes	No Diabetes	49609 (76.2)	1255 (76.3)
	Diabetes with complications	4783 (7.3)	110 (6.7)
	Diabetes without complications	10743 (16.5)	279 (17.0)
Obesity	No	53032 (88.1)	1380 (90.1)
	Yes	7167 (11.9)	151 (9.9)
Chronic Kidney Disease	No	55037 (82.2)	1389 (82.4)
	Yes	11927 (17.8)	296 (17.6)
Rheumatic Disease	No	58614 (88.5)	1506 (91.3)
	Yes	7614 (11.5)	143 (8.7)
Dementia	No	55348 (82.9)	1414 (84.7)
	Yes	11440 (17.1)	255 (15.3)

Table S2 – Overall characteristics of study cohort

	Total number of patients	Missing data		
Total N (%)				72179 (100.0)
Age on admission (years)	71987	192	Mean (SD)	70.2 (18.4)
Sex at Birth	71915	264	Male	40406 (56.2)
			Female	31509 (43.8)
Chronic cardiac disease	67454	4725	No	45388 (67.3)
			Yes	22066 (32.7)
Chronic kidney disease	66964	5215	No	55037 (82.2)
			Yes	11927 (17.8)
Chronic pulmonary disease (not asthma)	67171	5008	No	55152 (82.1)
			Yes	12019 (17.9)
Obesity (as defined by clinical staff)	60199	11980	No	53032 (88.1)
			Yes	7167 (11.9)
Diabetes	65135	7044	Diabetes with complications	4783 (7.3)
			Diabetes without complications	10743 (16.5)
			No Diabetes	49609 (76.2)
Rheumatologic disorder	66228	5951	No	58614 (88.5)
			Yes	7614 (11.5)
Dementia	66788	5391	No	55348 (82.9)
			Yes	11440 (17.1)

Table S3- Summary of matched imputed datasets for mortality outcome

		No NSAIDs	NSAIDs
Age (years)	<50	537 (12.77)	540 (12.84)
	50-69	1199 (28.51)	1198 (28.50)
	70-79	958 (22.78)	954 (22.69)
	80+	1512 (35.95)	1512 (35.97)
Sex at birth	Female	2256 (53.65)	2255 (53.63)
	Male	1949 (46.35)	1950 (46.37)
Chronic Cardiac Disease	No	2667 (63.42)	2668 (63.45)
	Yes	1538 (36.58)	1537 (36.55)
Diabetes Mellitus	No Diabetes	3196 (76.00)	3194 (75.96)
	Diabetes with complications	343 (8.16)	346 (8.23)
	Diabetes without complications	666 (15.84)	665 (15.81)
Obesity	No	3537 (84.11)	3536 (84.09)
	Yes	668 (15.89)	669 (15.91)
Chronic Kidney Disease	No	3402 (80.90)	3400 (80.86)
	Yes	803 (19.10)	805 (19.14)
Rheumatic disease	No	3339 (79.41)	3339 (79.41)
	Yes	866 (20.59)	866 (20.59)
Dementia	No	3571 (84.92)	3568 (84.85)
	Yes	634 (15.08)	637 (15.15)
Outcome (not included in matching)			
Death	Alive	2881 (68.51)	2932 (69.73)
	Died	1324 (31.49)	1273 (30.27)

Table S4- Summary of matched imputed datasets for critical care outcome

		No NSAIDs	NSAIDs
Age (years)	<50	533 (12.70)	537 (12.79)
	50-69	1200 (28.59)	1199 (28.55)
	70-79	954 (22.73)	954 (22.72)
	80+	1510 (35.98)	1509 (35.94)
Sex at birth	Male	2254 (53.69)	2253 (53.67)
	Female	1944 (46.31)	1945 (46.33)
Chronic Cardiac Disease	No	2660 (63.36)	2662 (63.41)
	Yes	1538 (36.64)	1536 (36.59)
Diabetes Mellitus	No Diabetes	3186 (75.89)	3186 (75.89)
	Diabetes with complications	341 (8.12)	344 (8.19)
	Diabetes without complications	671 (15.98)	668 (15.91)
Obesity	No	3528 (84.04)	3526 (83.99)
	Yes	670 (15.96)	672 (16.01)
Chronic Kidney Disease	No	3386 (80.66)	3386 (80.66)
	Yes	812 (19.34)	812 (19.34)
Rheumatic disease	No	3337 (79.49)	3337 (79.49)
	Yes	861 (20.51)	861 (20.51)
Dementia	No	3567 (84.97)	3562 (84.85)
	Yes	631 (15.03)	636 (15.15)
Outcome (not included in matching)			
Critical care admission	No	3604 (85.85)	3599 (85.73)
	Yes	594 (14.15)	599 (14.27)

Table S5- Summary of matched imputed datasets for invasive ventilation outcome

		No NSAIDs	NSAIDs
Age (years)	<50	529 (12.73)	533 (12.82)
	50-69	1190 (28.63)	1188 (28.59)
	70-79	945 (22.74)	946 (22.76)
	80+	1492 (35.90)	1489 (35.83)
Sex at birth	Male	2229 (53.63)	2227 (53.59)
	Female	1927 (46.37)	1929 (46.41)
Chronic Cardiac Disease	No	2639 (63.50)	2643 (63.59)
	Yes	1517 (36.50)	1513 (36.41)
Diabetes Mellitus	No Diabetes	3152 (75.84)	3153 (75.87)
	Diabetes with complications	344 (8.28)	344 (8.28)
	Diabetes without complications	660 (15.88)	659 (15.86)
Obesity	No	3490 (83.97)	3488 (83.93)
	Yes	666 (16.03)	668 (16.07)
Chronic Kidney Disease	No	3357 (80.77)	3356 (80.75)
	Yes	799 (19.23)	800 (19.25)
Rheumatic disease	No	3309 (79.62)	3308 (79.60)
	Yes	847 (20.38)	848 (20.40)
Dementia	No	3533 (85.01)	3528 (84.89)
	Yes	623 (14.99)	628 (15.11)
Outcome (not included in matching)			
Invasive mechanical ventilation	No	3805 (91.55)	3821 (91.94)
	Yes	351 (8.45)	335 (8.06)

Table S6- Summary of matched imputed datasets for non-invasive ventilation outcome

		No NSAIDs	NSAIDs
Age (years)	<50	528 (12.75)	532 (12.84)
	50-69	1186 (28.63)	1183 (28.56)
	70-79	945 (22.82)	945 (22.82)
	80+	1483 (35.80)	1482 (35.78)
Sex at birth	Male	2226 (53.74)	2224 (53.69)
	Female	1916 (46.26)	1918 (46.31)
Chronic Cardiac Disease	No	2627 (63.42)	2628 (63.45)
	Yes	1515 (36.58)	1514 (36.55)
Diabetes Mellitus	No Diabetes	3135 (75.69)	3133 (75.64)
	Diabetes with complications	341 (8.23)	344 (8.31)
	Diabetes without complications	666 (16.08)	665 (16.06)
Obesity	No	3479 (83.99)	3478 (83.97)
	Yes	663 (16.01)	664 (16.03)
Chronic Kidney Disease	No	3341 (80.66)	3341 (80.66)
	Yes	801 (19.34)	801 (19.34)
Rheumatic disease	No	3294 (79.53)	3294 (79.53)
	Yes	848 (20.47)	848 (20.47)
Dementia	No	3520 (84.98)	3516 (84.89)
	Yes	622 (15.02)	626 (15.11)
Outcome (not included in matching)			
Noninvasive ventilation	No	3496 (84.40)	3452 (83.34)
	Yes	646 (15.60)	690 (16.66)

Table S7- Summary of matched imputed datasets for supplemental oxygen outcome

		No NSAIDs	NSAIDs
Age (years)	<50	530 (12.77)	533 (12.84)
	50-69	1190 (28.67)	1187 (28.60)
	70-79	943 (22.72)	943 (22.72)
	80+	1487 (35.83)	1488 (35.85)
Sex at birth	Male	2237 (53.89)	2237 (53.89)
	Female	1914 (46.11)	1914 (46.11)
Chronic Cardiac Disease	No	2635 (63.48)	2635 (63.48)
	Yes	1516 (36.52)	1516 (36.52)
Diabetes Mellitus	No Diabetes	3146 (75.79)	3145 (75.76)
	Diabetes with complications	341 (8.21)	344 (8.29)
	Diabetes without complications	664 (16.00)	662 (15.95)
Obesity	No	3483 (83.91)	3482 (83.88)
	Yes	668 (16.09)	669 (16.12)
Chronic Kidney Disease	No	3354 (80.80)	3354 (80.80)
	Yes	797 (19.20)	797 (19.20)
Rheumatic disease	No	3295 (79.38)	3295 (79.38)
	Yes	856 (20.62)	856 (20.62)
Dementia	No	3534 (85.14)	3529 (85.02)
	Yes	617 (14.86)	622 (14.98)
Outcome (not included in matching)			
Supplemental oxygen	No	1419 (34.18)	1420 (34.21)
	Yes	2732 (65.82)	2731 (65.79)

Table S8- Summary of matched imputed datasets for acute kidney injury outcome

		No NSAIDs	NSAIDs
Age (years)	<50	420 (10.50)	423 (10.57)
	50-69	1170 (29.25)	1168 (29.20)
	70-79	936 (23.40)	936 (23.40)
	80+	1474 (36.85)	1473 (36.83)
Sex at birth	Male	2163 (54.07)	2161 (54.02)
	Female	1837 (45.92)	1839 (45.98)
Chronic Cardiac Disease	No	2506 (62.65)	2508 (62.70)
	Yes	1494 (37.35)	1492 (37.30)
Diabetes Mellitus	No Diabetes	3018 (75.45)	3019 (75.48)
	Diabetes with complications	332 (8.30)	333 (8.33)
	Diabetes without complications	650 (16.25)	648 (16.20)
Obesity	No	3352 (83.80)	3351 (83.78)
	Yes	648 (16.20)	649 (16.23)
Chronic Kidney Disease	No	3219 (80.47)	3217 (80.42)
	Yes	781 (19.53)	783 (19.57)
Rheumatic disease	No	3168 (79.20)	3167 (79.17)
	Yes	832 (20.80)	833 (20.82)
Dementia	No	3385 (84.62)	3380 (84.50)
	Yes	615 (15.38)	620 (15.50)
Outcome (not included in matching)			
Acute Kidney Injury	No	2980 (74.50)	2945 (73.62)
	Yes	1020 (25.50)	1055 (26.38)

Table S9 – Propensity matched secondary outcomes after excluding those who died

Outcome	Patients who were alive and had available data for matching (1:1)	Effect estimate (Odds ratio, 95%CI)
No NSAIDs (Reference)		1 (reference level)
Critical Care - NSAIDs	(3376:3376)	0.91 (0.79 to 1.06, p = 0.24)
Invasive ventilation - NSAIDs	(3350:3350)	0.84 (0.67 to 1.07, p = 0.15)
Non-invasive ventilation – NSAIDs	(3338:3338)	0.99 (0.80 to 1.22, p = 0.90)
Oxygen – NSAIDs	(3340:3340)	0.96 (0.85 to 1.08, p = 0.52)
AKI - NSAIDs	(2915:2915)	0.97 (0.84 to 1.13, p = 0.71)

AKI – Acute kidney injury, 95%CI – 95% confidence interval, NSAIDs – non-steroidal anti-inflammatory drugs

Table S10 – Effect of different NSAIDs on mortality in patients with COVID-19 after adjustment for explanatory variables in an unmatched cohort.

		Alive	Died	OR (univariable)	OR (multilevel)
NSAID type	No NSAIDs	42042 (67.4)	20372 (32.6)	-	-
	COX-2 Inhibitor	82 (75.9)	26 (24.1)	0.65 (0.41-1.00, p=0.060)	1.06 (0.64-1.77, p=0.82)
	Diclofenac/ Ketorolac /Naproxen	186 (68.1)	87 (31.9)	0.97 (0.74-1.24, p=0.79)	1.10 (0.81-1.50, p=0.53)
	Ibuprofen (or similar propionic acid derivative)	681 (74.9)	228 (25.1)	0.69 (0.59-0.80, p<0.0001)	0.88 (0.73-1.06, p=0.19)
	Mixed NSAIDs	551 (74.4)	190 (25.6)	0.71 (0.60-0.84, p=0.0001)	1.14 (0.93-1.39, p=0.20)
	Oxicam	91 (64.1)	51 (35.9)	1.16 (0.82-1.62, p=0.41)	1.25 (0.82-1.89, p=0.30)
Age on admission (years)	<50	9408 (94.7)	530 (5.3)	-	-
	50-69	15526 (79.5)	4008 (20.5)	4.58 (4.17-5.04, p<0.0001)	3.81 (3.38-4.30, p<0.0001)
	70-79	10181 (63.2)	5918 (36.8)	10.32 (9.41-11.34, p<0.0001)	8.14 (7.22-9.18, p<0.0001)
	80+	14395 (54.5)	12024 (45.5)	14.83 (13.55-16.25, p<0.0001)	12.14 (10.78-13.67, p<0.0001)
Sex at Birth	Female	22647 (71.9)	8862 (28.1)	-	-
	Male	26823 (66.4)	13583 (33.6)	1.29 (1.25-1.34, p<0.0001)	1.42 (1.36-1.48, p<0.0001)
Chronic cardiac disease	No	33429 (73.7)	11959 (26.3)	-	-
	Yes	12959 (58.7)	9107 (41.3)	1.96 (1.90-2.03, p<0.0001)	1.14 (1.09-1.19, p<0.0001)
Chronic pulmonary disease (not asthma)	No	39145 (71.0)	16007 (29.0)	-	-
	Yes	7128 (59.3)	4891 (40.7)	1.68 (1.61-1.75, p<0.0001)	1.24 (1.18-1.31, p<0.0001)
Chronic kidney disease	No	39354 (71.5)	15683 (28.5)	-	-
	Yes	6773 (56.8)	5154 (43.2)	1.91 (1.83-1.99, p<0.0001)	1.27 (1.21-1.34, p<0.0001)
Obesity (as defined by clinical staff)	No	36603 (69.0)	16429 (31.0)	-	-
	Yes	5194 (72.5)	1973 (27.5)	0.85 (0.80-0.89, p<0.0001)	1.21 (1.13-1.29, p<0.0001)
Diabetes	No Diabetes	35006 (70.6)	14603 (29.4)	-	-
	Diabetes with complications	3088 (64.6)	1695 (35.4)	1.32 (1.24-1.40, p<0.0001)	1.05 (0.97-1.14, p=0.19)
	Diabetes without complications	6899 (64.2)	3844 (35.8)	1.34 (1.28-1.40, p<0.0001)	1.19 (1.13-1.25, p<0.0001)
Rheumatologic disorder	No	40789 (69.6)	17825 (30.4)	-	-
	Yes	4922 (64.6)	2692 (35.4)	1.25 (1.19-1.32, p<0.0001)	0.94 (0.89-1.00, p=0.070)

NSAIDs – Nonsteroidal anti-inflammatory drugs, COX – Cyclooxygenase.

Table S11 - Sensitivity analysis of effect of Ibuprofen on in-hospital mortality, compared with no NSAIDs.

Dependent	Patients who were alive and had available data for matching (1:1)	Effect estimate (Odds ratio, 95%CI)
No NSAIDs (reference)		1 (reference level)
Ibuprofen	(721:721)	0.90 (0.71 to 1.13, p = 0.36)

NSAIDs – Nonsteroidal anti-inflammatory drugs, 95% CI – 95% Confidence Interval.

Table S12 – Sensitivity analysis of effect of Ibuprofen on in-hospital mortality, compared with other NSAIDs.

Outcome	Patients who were alive and had available data for matching (1:1)	Effect estimate (Odds ratio, 95%CI)
Other NSAIDs (reference)		1 (reference level)
Ibuprofen	(908:908)	0.82 (0.66 to 1.03, p = 0.082)

NSAIDs – Nonsteroidal anti-inflammatory drugs, 95% CI – 95% Confidence Interval.

Supplementary Figure 1 – Balance plots after imputation and propensity score matching

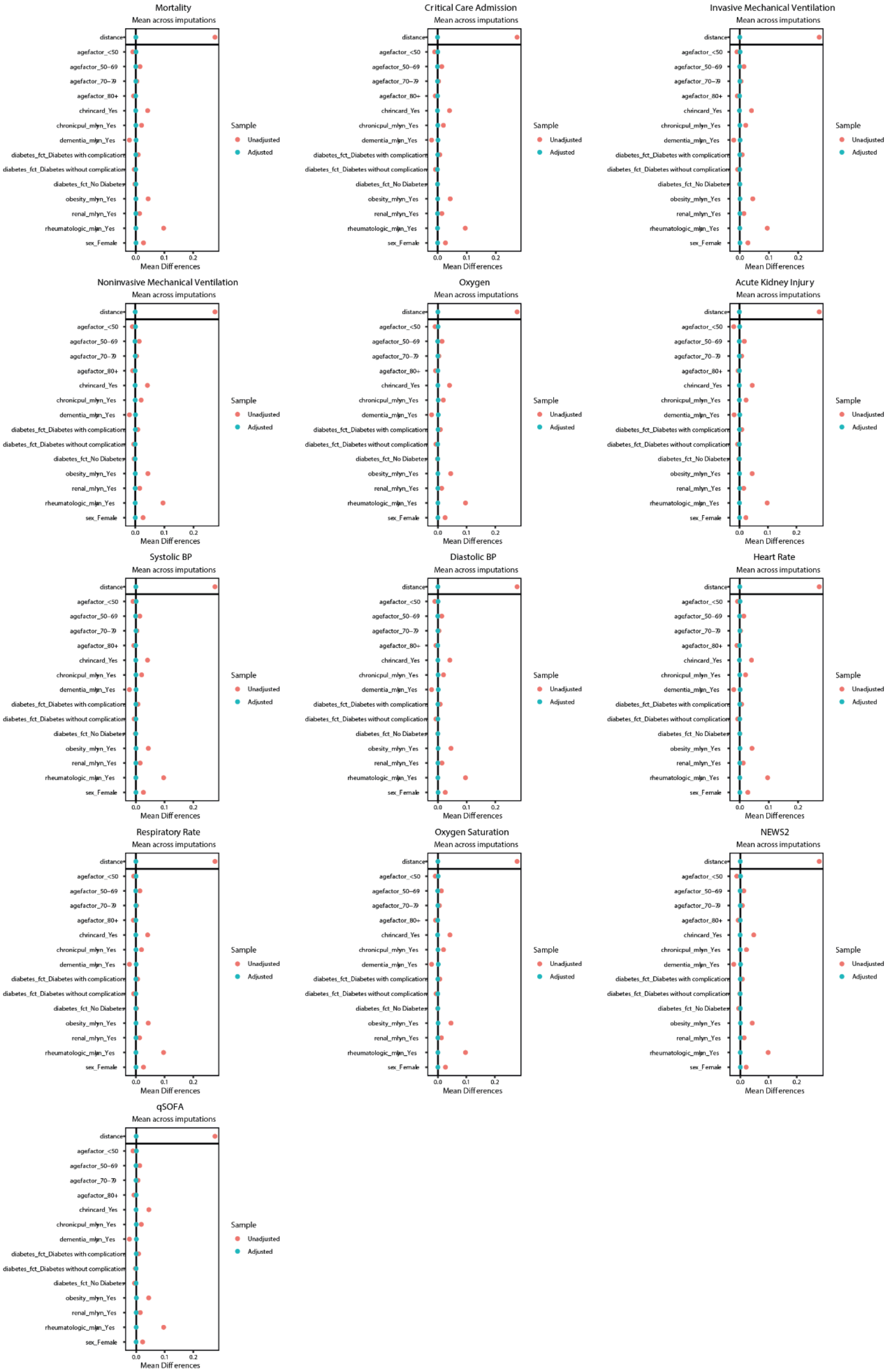

Supplementary Figure 2 – Outcomes in the unmatched cohort by age and NSAID use (y-axes scaled to reflect number of patients in each group).

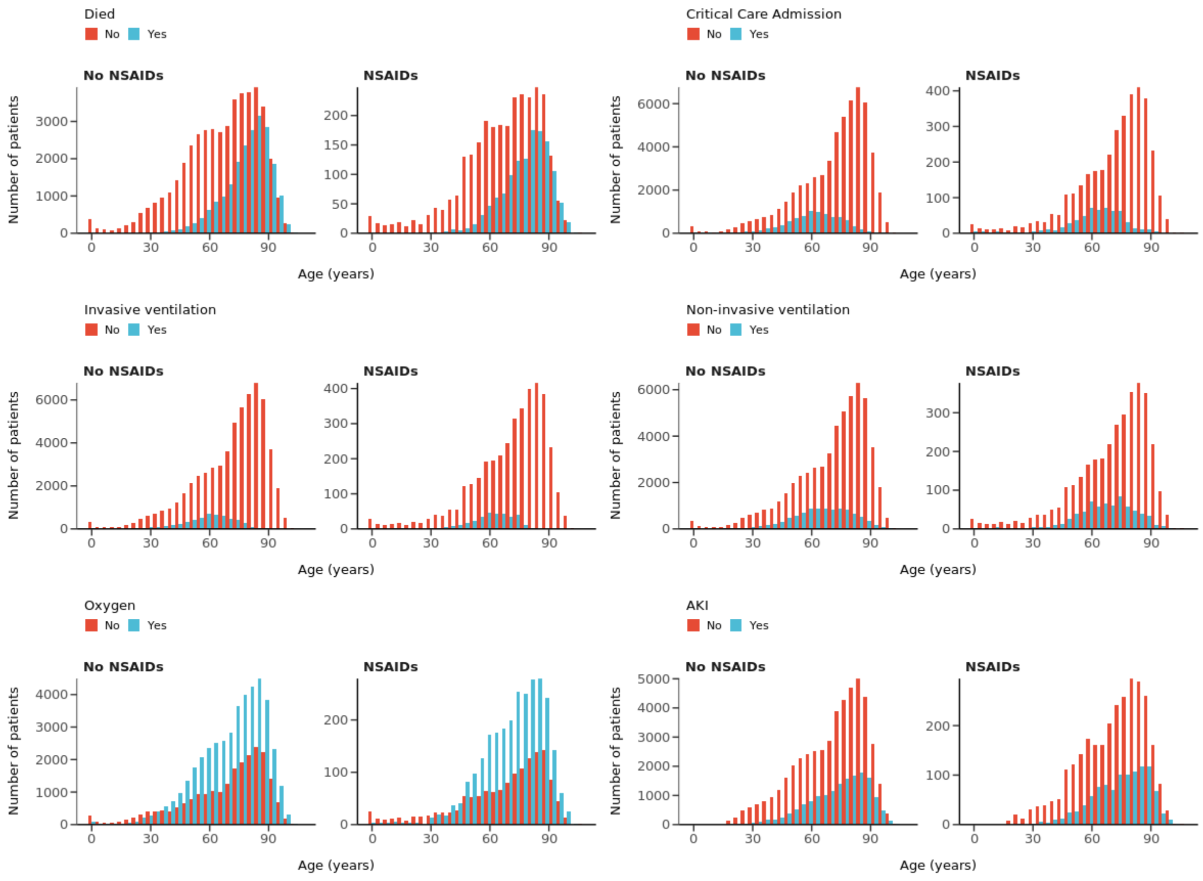

NSAIDs – Non-steroidal anti-inflammatory drugs.

Authorship supplement (for ISARIC4C investigators)

Consortium Lead Investigator: J Kenneth Baillie. Chief Investigator: Malcolm G Semple. Co-Lead Investigator: Peter JM Openshaw. ISARIC Clinical Coordinator: Gail Carson. Co-Investigator: Beatrice Alex, Benjamin Bach, Wendy S Barclay, Debby Bogaert, Meera Chand, Graham S Cooke, Annemarie B Docherty, Jake Dunning, Ana da Silva Filipe, Tom Fletcher, Christoper A Green, Ewen M Harrison, Julian A Hiscox, Antonia Ying Wai Ho, Peter W Horby, Samreen Ijaz, Saye Khoo, Paul Klenerman, Andrew Law, Wei Shen Lim, Alexander J Mentzer, Laura Merson, Alison M Meynert, Mahdad Noursadeghi, Shona C Moore, Massimo Palmarini, William A Paxton, Georgios Pollakis, Nicholas Price, Andrew Rambaut, David L Robertson, Clark D Russell, Vanessa Sancho-Shimizu, Janet T Scott, Thushan de Silva, Louise Sigfrid, Tom Solomon, Shiranee Sriskandan, David Stuart, Charlotte Summers, Richard S Tedder, Emma C Thomson, AA Roger Thompson, Ryan S Thwaites, Lance CW Turtle, Maria Zambon. Project Manager: Hayley Hardwick, Chloe Donohue, Ruth Lyons, Fiona Griffiths, Wilna Oosthuyzen. Data Analyst: Lisa Norman, Riinu Pius, Thomas M Drake, Cameron J Fairfield, Stephen R Knight, Kenneth A Mclean, Derek Murphy, Catherine A Shaw. Data and Information System Manager: Jo Dalton, Michelle Girvan, Egle Saviciute, Stephanie Roberts, Janet Harrison, Laura Marsh, Marie Connor, Sophie Halpin, Clare Jackson, Carrol Gamble. Data Integration and Presentation: Gary Leeming, Andrew Law, Murray Wham, Sara Clohisey, Ross Hendry, James Scott-Brown. Material Management: William Greenhalf, Victoria Shaw, Sara McDonald. Patient Engagement: Seán Keating. Outbreak Laboratory Staff and Volunteers: Katie A. Ahmed, Jane A Armstrong, Milton Ashworth, Innocent G Asiimwe, Siddharth Bakshi, Samantha L Barlow, Laura Booth, Benjamin Brennan, Katie Bullock, Benjamin WA Catterall, Jordan J Clark, Emily A Clarke, Sarah Cole, Louise Cooper, Helen Cox, Christopher Davis, Oslem Dincarslan, Chris Dunn, Philip Dyer, Angela Elliott, Anthony Evans, Lorna Finch, Lewis WS Fisher, Terry Foster, Isabel Garcia-Dorival, William Greenhalf, Philip Gunning, Catherine Hartley, Rebecca L Jensen, Christopher B Jones, Trevor R Jones, Shadia Khandaker, Katharine King, Robyn T. Kiy, Chrysa Koukorava, Annette Lake, Suzannah Lant, Diane Latawiec, Lara Lavelle-Langham, Daniella Lefteri, Lauren Lett, Lucia A Livoti, Maria Mancini, Sarah McDonald, Laurence McEvoy, John McLauchlan, Soeren Metelmann, Nahida S Miah, Joanna Middleton, Joyce Mitchell, Shona C Moore, Ellen G

Murphy, Rebekah Penrice-Randal, Jack Pilgrim, Tessa Prince, Will Reynolds, P. Matthew Ridley, Debby Sales, Victoria E Shaw, Rebecca K Shears, Benjamin Small, Krishanthi S Subramaniam, Agnieska Szemiel, Aislynn Taggart, Jolanta Tanianis-Hughes, Jordan Thomas, Erwan Trochu, Libby van Tonder, Eve Wilcock, J. Eunice Zhang, Lisa Flaherty, Nicole Maziere, Emily Cass, Alejandra Doce Carracedo, Nicola Carlucci, Anthony Holmes, Hannah Massey. Edinburgh Laboratory Staff and Volunteers: Lee Murphy, Nicola Wrobel, Sarah McCafferty, Kirstie Morrice, Alan MacLean. Local Principal Investigators: Kayode Adeniji, Daniel Agranoff, Ken Agwuh, Dhiraj Ail, Erin L. Aldera, Ana Alegria, Brian Angus, Abdul Ashish, Dougal Atkinson, Shahedal Bari, Gavin Barlow, Stella Barnass, Nicholas Barrett, Christopher Bassford, Sneha Basude, David Baxter, Michael Beadsworth, Jolanta Bernatoniene, John Berridge, Nicola Best, Pieter Bothma, David Chadwick, Robin Brittain-Long, Naomi Bulteel, Tom Burden, Andrew Burtenshaw, Vikki Caruth, David Chadwick, Duncan Chambler, Nigel Chee, Jenny Child, Srikanth Chukkambotla, Tom Clark, Paul Collini, Catherine Cosgrove, Jason Cupitt, Maria-Teresa Cutino-Moguel, Paul Dark, Chris Dawson, Samir Dervisevic, Phil Donnison, Sam Douthwaite, Ingrid DuRand, Ahilanadan Dushianthan, Tristan Dyer, Cariad Evans, Chi Eziefula, Chrisopher Fegan, Adam Finn, Duncan Fullerton, Sanjeev Garg, Sanjeev Garg, Atul Garg, Effrossyni Gkrania-Klotsas, Jo Godden, Arthur Goldsmith, Clive Graham, Elaine Hardy, Stuart Hartshorn, Daniel Harvey, Peter Havalda, Daniel B Hawcutt, Maria Hobrok, Luke Hodgson, Anil Hormis, Michael Jacobs, Susan Jain, Paul Jennings, Agilan Kaliappan, Vidya Kasipandian, Stephen Kegg, Michael Kelsey, Jason Kendall, Caroline Kerrison, Ian Kerslake, Oliver Koch, Gouri Koduri, George Koshy, Shondipon Laha, Steven Laird, Susan Larkin, Tamas Leiner, Patrick Lillie, James Limb, Vanessa Linnett, Jeff Little, Mark Lyttle, Michael MacMahon, Emily MacNaughton, Ravish Mankregod, Huw Masson, Elijah Matovu, Katherine McCullough, Ruth McEwen, Manjula Meda, Gary Mills, Jane Minton, Mariyam Mirfenderesky, Kavya Mohandas, Quen Mok, James Moon, Elinoor Moore, Patrick Morgan, Craig Morris, Katherine Mortimore, Samuel Moses, Mbiye Mpenge, Rohinton Mulla, Michael Murphy, Megan Nagel, Thapas Nagarajan, Mark Nelson, Matthew K. O'Shea, Igor Otahal, Marlies Ostermann, Mark Pais, Selva Panchatsharam, Danai Papakonstantinou, Hassan Paraiso, Brij Patel, Natalie Pattison, Justin Pepperell, Mark Peters, Mandeep Phull, Stefania Pintus, Jagtur Singh Pooni, Frank Post, David Price, Rachel Prout, Nikolas

Rae, Henrik Reschreiter, Tim Reynolds, Neil Richardson, Mark Roberts, Devender Roberts, Alistair Rose, Guy Rousseau, Brendan Ryan, Taranprit Saluja, Aarti Shah, Prad Shanmuga, Anil Sharma, Anna Shawcross, Jeremy Sizer, Manu Shankar-Hari, Richard Smith, Catherine Snelson, Nick Spittle, Nikki Staines, Tom Stambach, Richard Stewart, Pradeep Subudhi, Tamas Szakmany, Kate Tatham, Jo Thomas, Chris Thompson, Robert Thompson, Ascanio Tridente, Darell Tupper-Carey, Mary Twagira, Andrew Ustianowski, Nick Vallotton, Lisa Vincent-Smith, Shico Visuvanathan, Alan Vuylsteke, Sam Waddy, Rachel Wake, Andrew Walden, Ingeborg Welters, Tony Whitehouse, Paul Whittaker, Ashley Whittington, Padmasayee Papineni, Meme Wijesinghe, Martin Williams, Lawrence Wilson, Sarah Cole, Stephen Winchester, Martin Wiselka, Adam Wolverson, Daniel G Wooton, Andrew Workman, Bryan Yates, Peter Young.
